# Leveraging Multiactions to Improve Medical Personalized Ranking for Collaborative Filtering

**DOI:** 10.1155/2017/5967302

**Published:** 2017-10-03

**Authors:** Shan Gao, Guibing Guo, Runzhi Li, Zongmin Wang

**Affiliations:** ^1^School of Information Engineering, Zhengzhou University, Zhengzhou 450001, China; ^2^College of Information Science and Engineering, Henan University of Technology, Zhengzhou 450001, China; ^3^Collaborative Innovation Center for Internet Healthcare, Zhengzhou University, Zhengzhou 450052, China; ^4^Software College, Northeastern University, Shenyang 110169, China

## Abstract

Nowadays, providing high-quality recommendation services to users is an essential component in web applications, including shopping, making friends, and healthcare. This can be regarded either as a problem of estimating users' preference by exploiting explicit feedbacks (numerical ratings), or as a problem of collaborative ranking with implicit feedback (e.g., purchases, views, and clicks). Previous works for solving this issue include pointwise regression methods and pairwise ranking methods. The emerging healthcare websites and online medical databases impose a new challenge for medical service recommendation. In this paper, we develop a model, MBPR (Medical Bayesian Personalized Ranking over multiple users' actions), based on the simple observation that users tend to assign higher ranks to some kind of healthcare services that are meanwhile preferred in users' other actions. Experimental results on the real-world datasets demonstrate that MBPR achieves more accurate recommendations than several state-of-the-art methods and shows its generality and scalability via experiments on the datasets from one mobile shopping app.

## 1. Introduction

With the continuous improvement of people's living standards, healthcare has attracted more and more attention and becomes a hot research topic. The phenomenon of scarcity and unbalanced distribution of medical resources across areas in China becomes a serious social problem. Under current circumstances, it is quite difficult for people to choose appropriate hospitals and doctors. The main channels that patients obtain healthcare information include recommendations from other people by word-of-mouth, advertisements on newspapers or television, and more often in the last decade, using search engines on the Internet (Baidu, Google, etc.). Unfortunately, these methods cannot ensure information quality, accuracy, and reliability of acquaintances' recommendations. Given the importance and seriousness of people's wellbeing, people always go to reputed general hospitals for medical requirements, which lead to the phenomenon of overcapacity in AAA grade comprehensive hospitals and under capacity in Community Health Service Institutions. It forms a trend that people prefer high-reputation hospitals, and thus it worsens the unbalance of medical resources. For the patients, without professional knowledge and relevant medical experience, they spend more unnecessary time and energy in this scenario. Given the expensive healthcare expenses, it is in danger of making the wrong judgments and giving up their medical treatment.

Therefore, it is a vital issue to help patients to attend an appropriate level of medical resource. As emerging medical databases and websites provide tremendous information, a personalized healthcare recommendation service based on web mining methods can be devised. *MedHelp* (http://www.medhelp.org/) is an online health community, which offers tracking tools for pain, weight, and other chronic conditions. Patients will receive guidance, motivation, and support from peers and experts. *CureTogether* (http://curetogether.com/) is a website where people anonymously talk about sensitive symptoms, compare health data to better analyze their health status, and receive more informative treatment decisions and new research discoveries based on patient-contributed data. People can choose medical service from other health-related review websites, such as *Vitals* (http://www.vitals.com/), *Healthgrades* (http://www.healthgrades.com/), and *RateMDs* (http://www.ratemds.com/). On these websites, detailed information about hospitals and doctors' online appointment service can be obtained. This innovative process of medical consultation improves efficiency compared to traditional onsite doctor selection [1].

Recommender systems can help users deal with the *information overload* problem efficiently by suggesting items (e.g., products, movie, and music) that match users' personal preference [2, 3]. Collaborative filtering [4], a widely exploited technique, has been extensively adopted in commercial recommender systems [5–7]. In previous works, model-based methods have been proposed to improve the predictive accuracy using explicit feedbacks (e.g., numerical ratings) [8–10]. However, in many real application scenarios, explicit numerical ratings might not be available. Some recent works turn to improve the recommendation performance via exploiting users' implicit feedback, such as browsing [11], clicking [5], watching [6], and purchasing [12]. This is known as the one-class recommendation problem, and various solutions have been proposed to solve it by making use of auxiliary relations (e.g., social information).

MR-BPR [13], a state-of-the-art method treating one-class recommendation as a multirelational learning problem, focuses on how to make use of social information on users for item prediction and presents an extension of Bayesian Personalized Ranking for multirelational ranking in social networks. In this work, MR-BPR models users' social preference and item preference simultaneously, but it fails to model how auxiliary relations (i.e., social relations) directly influence users' preferences on items. Zhao et al. develop SBPR [14], to model user preference ranking of items by utilizing the social connections from users' friends. In [14], a new social feedback class by exploiting users' social information is introduced, and the parameter of social coefficient can indicate the attitude from users' social relations towards an item. However, the social feedback is only based on the users' social information with their friends, and this type of feedback can also be considered the “negative feedback.” Nevertheless, few works have adopted multiple kinds of observed feedback coming from multiactions between the users and the items simultaneously for the one-class recommendation problem, especially in healthcare recommendation.

In this paper, we study how to leverage multiple observed feedback for better recommendation models, given the assumption regarding a new class of items referred to as “auxiliary feedback.” And, a special coefficient is introduced to indicate the preference distance between multiple actions of the users. We then propose a new algorithm called Medical Bayesian Personalized Ranking over multiple users' actions (MBPR). The proposed method is evaluated on a real-world dataset which is collected from a healthcare service website, and empirical results show that the model is more effective and can achieve better recommendation performance. The generality of our approach is also demonstrated in the experiments by being applied to another dataset from mobile e-commence application.

## 2. Related Works

In this section, we will briefly review some related works in two aspects: (1) methods based on pointwise preference assumptions and (2) methods based on pairwise preference assumptions.

In pointwise methods, the implicit feedback is taken as absolute preference scores. Specifically, an observed user-item pair {*u*, *i*} is regarded as a positive feedback and interpreted as that user *u* likes item *i* with a high absolute score. The negative feedback is sampled as low preference scores using several strategies. The two typical pointwise approaches for solving this recommendation problem are OCCF (one-class collaborative filtering) [15] and iMF (implicit matrix factorization) [16], where matrix factorization methods can be applied to these methods. OCCF [15] proposes two different sampling strategies for unobserved user-item interactions to solve the one-class recommendation problem. One is weighted low-rank approximation; the other is negative example sampling. In iMF [16] work, confidence weights on implicit feedback is introduced, which can be approximated by two latent feature matrices. However, the limitation of OCCF is that the unobserved user-item pairs are taken as a negative feedback and unobserved user-item pairs {*u*, *j*} do not always indicate that user *u* dislikes item *j* in real world. As for iMF, the auxiliary knowledge of confidence is required for each observed feedback, which may not be available in real applications.

Compared with pointwise methods, pairwise methods take implicit feedback as relative preferences rather than absolute ones, and the order or ranking of the feedback is focused on. For example, the user-item-item triple {*u*, *i*, *j*} indicates that user *u* is assumed to prefer item *i* over item *j*, which can be interpreted as this user shows higher preference on the positive feedback than on the negative feedback. In [12], Bayesian Personalized Ranking (BPR) algorithm is firstly proposed with such pairwise preference assumption for solving the one-class collaborative filtering problem. Following this framework, various new works have been proposed to combine different types of contextual data into the BPR algorithm. Pan and Chen [11] develop a general algorithm called collaborative filtering via learning pairwise preferences over item sets (CoFiSet) based on a new and relaxed assumption of pairwise preferences over item sets, which defines a user's preference on a set of items (item set) instead of on a single item. Du et al. [17] propose a novel method called User Graph regularized Pairwise Matrix Factorization (UGPMF), to improve recommendation performance by incorporating user-side social connections into the pairwise matrix factorization procedure. Pan and Chen [18] propose an improved assumption and group Bayesian Personalized Ranking (GBPR), via introducing a new concept of group preference to relax the two fundamental assumptions made in the pairwise ranking methods. This algorithm uses richer interactions among users and aggregates the features of a group of related users. Zhao et al. [14] design a pairwise algorithm called Social Bayesian Personalized Ranking (SBPR) which is based on the simple observation that users tend to assign higher ranks to items that their friends prefer, and this method uses social connections to better estimate users' rankings of products. Rendle and Freudenthaler [19] propose a nonuniform and context-dependent item sampler of negative items via oversampling informative pairs to speed up convergence.

However, the aforementioned works mainly focus on modeling the feedback order by using users' positive feedback, negative feedback, or social information, but do not investigate how the feedback from users' other actions can be combined to model users' preference order on items. Compared with these methods, our proposed MBPR algorithm exploits two kinds of observed feedback indicating multiple actions of the users in order to build better models of users' preferences.

## 3. Problem Definition

In this section, we will first introduce the dataset which is collected from a healthcare service website (*Topmd* (http://www.topmd.cn/)). And then, we will present the basic concepts and definitions used in the paper and elaborate the problem of Medical Bayesian Personalized Ranking over multiple users' actions.

Let *U* = {*u*}_*u*=1_^*m*^ denote the user sets, *I* = {*i*}_*i*=1_^*n*^ denote the item sets, *u* ∈ *U*, *i*, *k*, *j* ∈ *I*.

**Figure 1:**
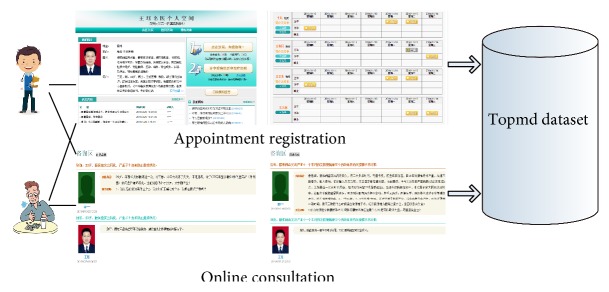
Topmd dataset.

The website *Topmd* is designed and developed by the laboratory which the author works in. The users' main actions include Appointment Registration and Online Consultation with the doctors which are enrolled formally in this website. In this situation, the “doctors” can be defined as the “items.” The numbers of user *u* made an appointment to doctor *i* or user *u* consulted doctor *k* are added up separately. “Positive Feedback” in the dataset represents whether users made an appointment with a doctor, and “Auxiliary Feedback” represents whether users consulted a doctor on the website. The Topmd dataset is briefly illustrated in Figure 1. In this paper, these two kinds of observed feedback coming from multiple users' actions are exploited simultaneously to improve the recommendation performance.

The concepts that will be used in this paper are defined as the following.

### 3.1. Observed Items and Unobserved Items

For each user *u* ∈ *U*, observed items *FA*_*u*_ ∈ *I* and *FC*_*u*_ ∈ *I* include the items which user *u* shows two different kinds of observed preference, respectively. Unobserved items ${\bar{F}}_{u}\in I$ are the remaining items. In this work, for each user *u* ∈ *U*, we divide the total item set *I* into three parts: positive feedback, auxiliary feedback, and negative feedback, just as follows.

#### 3.1.1. Positive Feedback

Positive feedback *P*_*u*_ = {(*u*, *i*)} is defined as the set of user-item pairs containing user *u* and his/her observed items *i* ∈ *FA*_*u*_.These could be the items that user *u* purchased, rated, reviewed, and so forth. According to the dataset in question, *P_u_* is defined as the item sets (i.e., doctors) that have been made an appointment by user *u*.

#### 3.1.2. Auxiliary Feedback

Auxiliary feedback *AP*_*u*_ = {(*u*, *k*)} is defined as the set of user-item pairs containing user *u* and his observed items *k* ∈ *FC*_*u*_. According to the dataset in question, *AP*_*u*_ is defined as the item sets (i.e., doctors) that have been consulted online by user *u*.

#### 3.1.3. Negative Feedback

*N*_*u*_ = {(*u*, *j*)} indicates negative feedback defined as the set of user-item pairs, where $j\in {\bar{F}}_{u}$ represents items that user *u* has neither made an appointment nor consulted. Note that a negative feedback does not represent that a user dislikes the items.

It is obvious that *P*_*u*_∩*AP*_*u*_∩*N*_*u*_ = ∅ and *P*_*u*_ ∪ *AP*_*u*_ ∪ *N*_*u*_ include all the item sets.

### 3.2. Auxiliary Coefficient

Given the definition of auxiliary feedback, we introduce an auxiliary coefficient *m*_*uik*_ which describes the preference distance between *u*'s positive feedback and auxiliary feedback. Given a particular user *u*, associated with their positive feedback {(*u*, *i*)} ∈ *P*_*u*_ and auxiliary feedback {(*u*, *k*)} ∈ *AP*_*u*_, *m*_*uik*_ is a parameter indicating the preference distance between *u*'s positive feedback towards item *i* and auxiliary feedback towards a particular item *k*. The value and the computational method of the auxiliary coefficient will be discussed later. It can be found that the larger the value of the auxiliary coefficient, the bigger the preference distance between the appointment action and consultation action. In this situation, we can naturally assume that user *u* may also make an appointment to item *k* which was only observed in auxiliary feedback.

We list some notations used in the paper in Table 1.

Unlike the previous works, we introduce a new auxiliary feedback class by exploiting users' other kind of action information. With these concepts, the problem of Medical Bayesian Personalized Ranking over multiple users' actions can be defined. The goal of this paper is to recommend a personalized ranked list of items for each user *u*. According to the above concepts which are defined using both user positive feedback and auxiliary feedback, the main task is how to learn a ranking function that incorporates all of these sources of information.

The problem of leveraging auxiliary feedback (i.e., healthcare consultation information) to improve personalized ranking for collaborative filtering can be defined precisely as follows:

Given observed feedback *S*^Train^ = (*U*, *I*) and the auxiliary feedback coming from multiple actions, the target of this paper is to learn a ranking function for each user *u*.

(1) $$\begin{array}{c} f:\left(u,S^{Train},P_{u},{AP}_{u},N_{u},\left\{m_{uik}\right\}\right)\to Ranked\_list\left(I\right)\text{: }r_{1}\left(m\right)≻\ldots r_{i}\left(p\right)≻r_{i+1}\left(q\right)\ldots , \end{array}$$

where *r*_*i*_(*p*)≻*r*_*i*+1_(*q*) represents that user *u* shows higher preference towards item *p* than item *q*.

## 4. Medical Bayesian Personalized Ranking over Multiple Users' Actions

In this section, we will describe our model assumption regarding positive, auxiliary, and negative feedbacks and then detail the proposed algorithm of Medical Bayesian Personalized Ranking over multiple users' actions.

Unlike the previous works, we incorporate auxiliary feedback from a user's healthcare consult information and introduce a coefficient based on the preference distance between positive feedback and auxiliary feedback that controls how training pairs are sampled.

### 4.1. Model Assumption

We firstly introduce the basic assumption adopted by the Bayesian Personalized Ranking (BPR) [12]. BPR's main idea is to use partial order of items, instead of single user-item examples, to train a recommendation model, which can be represented as

(2) $$\begin{array}{c} x_{ui}≻x_{uj},\;i\in P_{u},j\in N_{u}, \end{array}$$

where *x*_*ui*_ represents the preference of user *u* on item *i*. Given a positive user-item example of user *u* on item *i* (e.g., user *u* viewed or purchased item *i*), we assume that the user likely prefers the item *i* ∈ *P*_*u*_ to all other nonobserved items *j* ∈ *N*_*u*_.This relation is expressed by *x*_*ui*_≻*x*_*uj*_. The differences between the basic idea of point-wise and pairwise can be reflected by this assumption. Point-wise methods [15, 16] focus on fitting the numeric rating values whereas pairwise methods [12, 20, 21] model the preference order of the data instead, which can extract a pairwise preference dataset *D* : *U* × *I* × *I* by

(3) $$\begin{array}{c} D≔\left\{\left(u,i,j\right)\;|\;i\in I_{u}^{+}\wedge j\in I\backslash I_{u}^{+}\right\}, \end{array}$$

where *I*_*u*_^+^ is the positive item set and *I*\*I*_*u*_^+^ is the missing set associated with user *u*. The semantics of each triple (*u*, *i*, *j*) ∈ *D* is that user *u* is assumed to prefer item *i* over item *j*.

The target of the optimization criterion for personalized ranking BPR-OPT is to maximize the following posterior probability over these pairs:

(4) $$\begin{array}{c} BPR-Opt≔\sum_{\left(u,i,j\right)\in D}\text{ln}\sigma \left({\hat{x}}_{uij}\right)-\lambda_{\theta }\left(\theta \right), \end{array}$$

where *σ*(*x*) is the logistic sigmoid function

(5) $$\begin{array}{c} \sigma \left(x\right)≔\frac{1}{1+e^{-x}}. \end{array}$$

The *θ* represents the parameter vector of an arbitrary model class (e.g., matrix factorization), and *λ*_*θ*_ is model-specific regularization parameters.

Previous works have shown that the pairwise assumption generates better recommendation results than the pointwise methods. Now, our proposed assumption is detailed based on the following pairwise preference comparisons.

There are many kinds of medical services under the circumstances of healthcare recommendation. Based on the datasets collected from the healthcare website, we select the most representative two types of users' behaviors. One is the appointment registration, and the other is online health consultation. Given this profile, the assumptions are proposed just like as follows:

(6) $$\begin{array}{c} x_{ui}≻x_{uk},x_{uk}≻x_{uj},\;i\in P_{u},k\in {AP}_{u},j\in N_{u}, \end{array}$$

where *x*_*ui*_ represents user *u*'s preference on positive feedback *i*, *x*_*uk*_ represents the preference on auxiliary feedback *k*, and *x*_*uj*_ represents the preference on negative feedback *j*. Based on this assumption, the “observed” feedback is composed of two parts: positive feedback and auxiliary feedback. According to the application scenario of the dataset, the positive feedback is the set of user-item pairs coming from the reservation relationship, and the auxiliary feedback is the set of user-item pairs according to the health consultation relationship. The proposed assumption considers both the influence of a user's positive feedback as well as their auxiliary feedback, making it more general and realistic in real medical recommendation settings.

### 4.2. Model Formulation

In this section, we will introduce the formulation and learning of the model with the assumption as in (6), and the experimental comparison will be described in Section 5.

For each user, the optimization criterion can be represented as follows:

(7) $$\begin{array}{c} \prod_{i,k\in {PAP}_{u}}p{\left(x_{ui}≻x_{uk}\;|\;\theta \right)}^{\delta \left(u,i,k\right)}{\left[1-p\left(x_{ui}≻x_{uk}\;|\;\theta \right)\right]}^{1-\delta \left(u,i,k\right)},\\ \prod_{k,j\in {APN}_{u}}p{\left(x_{uk}≻x_{uj}\;|\;\theta \right)}^{\tau \left(u,k,j\right)}{\left[1-p\left(x_{uk}≻x_{uj}\;|\;\theta \right)\right]}^{1-\tau \left(u,k,j\right)}, \end{array}$$

where *PAP*_*u*_ = *P*_*u*_ ∪ *AP*_*u*_, *APN*_*u*_ = *AP*_*u*_ ∪ *N*_*u*_, and *δ*(*u*, *i*, *k*) and *τ*(*u*, *k*, *j*) are the indicator function

(8) $$\begin{array}{c} \delta \left(u,i,k\right)≔\left\{\begin{array}{l} 1,\;i\in P_{u},\;k\in AP\\ 0,\;else, \end{array}\right.\\ \tau \left(u,k,j\right)≔\left\{\begin{array}{l} 1,\;k\in {AP}_{u},\;j\in N_{u}\\ 0,\;else. \end{array}\right. \end{array}$$

For a specific user of the data set, (7) reflects the main assumption proposed in Section 4.1 of this paper. On the one hand, the user's preference due to positive feedback from the reservation actions should be larger than that of auxiliary feedback from health consultation, and on the other hand his preference due to auxiliary feedback should be larger than that of negative feedback.

Due to the totality and antisymmetry of a pairwise ordering scheme as detailed in [12], the (7) can be rewritten as

(9) $$\begin{array}{c} \frac{\sum_{i\in P_{u},k\in {AP}_{u}}p\left(x_{ui}≻x_{uk}\;|\;\theta \right)}{\left|P_{u}\right|\left|{AP}_{u}\right|}+\frac{\sum_{k\in {AP}_{u},j\in N_{u}}p\left(x_{uk}≻x_{uj}\;|\;\theta \right)}{\left|{AP}_{u}\right|\left|N_{u}\right|}. \end{array}$$

With this assumption, we have a new criterion called Medical Bayesian Personalized Ranking over multiple users' actions (MBPR). Our goal is to maximize the following objective function:

(10) $$\begin{array}{c} \sum_{u}\left[\sum_{i\in P_{u}}\sum_{k\in {AP}_{u}}\text{ln}\left(\sigma \left(\frac{x_{ui}-x_{uk}}{1+e^{-m_{uik}}}\right)\right)+\sum_{k\in {AP}_{u}}\sum_{j\in N_{u}}\text{ln}\left(\sigma \left(x_{uk}-x_{uj}\right)\right)\right]-regularization, \end{array}$$

where a regularization term is used to prevent overfitting.

### 4.3. Auxiliary Coefficient

Unlike other works, the coefficient *m*_*uik*_ is employed in (10) to control the contribution of each sampled training pair to the objective function. This coefficient indicates the preference distance between positive feedback and auxiliary feedback. Auxiliary feedback with a large auxiliary coefficient implies that items have a higher probability of being adopted or preferred by users. In our dataset based on healthcare service, the frequency of a user making an appointment or health counselling is believed to be the significant evaluation index, which can indicate the preference of the user to the item (i.e., doctors). And so, we will detail the computation method of this coefficient on the basis of the specific circumstances.

#### 4.3.1. The First Method

We define *t*_*ui*_ as the number which user *u* has made to item *i* based on one kind action and *t*_*uk*_ as the number which user *u* has made to item *j* based on auxiliary action. According to the dataset which is collected from a real-life scenario, the positive feedback is the set of user-item pairs based on the reservation action, and the auxiliary feedback is the set of user-item pairs coming from the health consultation action. *t*_*ui*_ is the number that user *u* has made an appointment to item *i*, and *t*_*uk*_ is the number that user *u* has counselled item *k*. By comparison, the frequency of a user making an appointment to the frequency of health counselling, there are two kinds of situations as follows:

1. If *t*_*ui*_ ≥ *t*_*uk*_, and then *t*_*ui*_ − *t*_*uk*_ ≥ 0, the larger the difference between *t*_*ui*_ and *t*_*uk*_, the bigger the user *u*'s preference for item *i* than item *k*.
2. If *t*_*ui*_ < *t*_*uk*_, and then *t*_*ui*_ − *t*_*uk*_ < 0, the smaller the difference between *t*_*ui*_ and *t*_*uk*_, the smaller the difference between *u*'s preference for item *i* than item *k*.

And thus, the auxiliary coefficient can be defined as

(11) $$\begin{array}{c} m_{uik}≔t_{ui}-t_{uk}. \end{array}$$

Based on the above analysis, the auxiliary coefficient can be computed with the logistic sigmoid function

(12) $$\begin{array}{c} \sigma \left(m_{uik}\right)≔\frac{1}{1+e^{-m_{uik}}}. \end{array}$$

And (10) can be rewritten as

(13) $$\begin{array}{c} \sum_{u}\left[\sum_{i\in P_{u}}\sum_{k\in {AP}_{u}}\text{ln}\left(\sigma \left(\frac{x_{ui}-x_{uk}}{1+e^{-\left(t_{ui}-t_{uk}\right)}}\right)\right)+\sum_{k\in {AP}_{u}}\sum_{j\in N_{u}}\text{ln}\left(\sigma \left(x_{uk}-x_{uj}\right)\right)\right]-\lambda_{\theta }{\left\|\theta \right\|}^{2}. \end{array}$$

#### 4.3.2. The Second Method

The auxiliary coefficient *m*_*uik*_ can be regarded as one of the model parameters. Firstly, the initial value of *m*_*uik*_ can be assigned by (11) and then is iteratively updated based on the sampled feedback pairs using

(14) $$\begin{array}{c} \nabla m_{uik}=\frac{\partial f\left(\theta \right)}{\partial m_{uik}}, \end{array}$$

(15) $$\begin{array}{c} m_{uik}=m_{uik}-\gamma \nabla m_{uik}, \end{array}$$

where *γ* > 0 is the learning rate.

Based on the two methods described previously, the experiments will be conducted and the comparative analysis will be demonstrated in Section 5.

### 4.4. Model Learning

The optimization problem described in (13) can be solved by adopting the widely used stochastic gradient descent (SGD) algorithm in collaborative filtering [16]. The main process of SGD is to randomly select a ((positive, auxiliary) and (auxiliary, negative)) feedback pair, and then the model parameters are iteratively updated based on the sampled feedback pairs. We will firstly derive the gradients and update rules for each variable.

In our work, the model of matrix factorization is used in modeling the hidden preferences of a user on an item for the preference function, *x*_*ui*_ = *W*_*u*_^*T*^*H*_*i*_ + *b*_*i*_, *x*_*uk*_ = *W*_*u*_^*T*^*H*_*k*_ + *b*_*k*_, *x*_*uj*_ = *W*_*u*_^*T*^*H*_*j*_ + *b*_*j*_, *W* ∈ *R*^*d*×*m*^, *H* ∈ *R*^*d*×*n*^, and *b* ∈ *R*^*n*^, where *d* is the number of latent factors and *θ* = {*W*, *H*, *b*} are the model parameters for matrix factorization.

According to (13), the regularization term can be rewritten as

(16) $$\begin{array}{c} \lambda_{\theta }{\left\|\theta \right\|}^{2}=\lambda {\left\|W_{u\cdot }\right\|}^{2}+\lambda {\left\|H_{i\cdot }\right\|}^{2}+\lambda {\left\|H_{k\cdot }\right\|}^{2}+\lambda {\left\|H_{j\cdot }\right\|}^{2}+\lambda {\left\|b_{i}\right\|}^{2}+\lambda {\left\|b_{k}\right\|}^{2}+\lambda {\left\|b_{j}\right\|}^{2}. \end{array}$$

We have the gradients of the variables including the loss term and the regularization term

(17) $$\begin{array}{c} \nabla W_{u\cdot }=\frac{\partial f\left(\theta \right)}{\partial W_{u\cdot }}=\sigma \left(\frac{x_{ui}-x_{uk}}{1+e^{-m_{uik}}}\right)\times \left(H_{i\cdot }-H_{k\cdot }\right)+\sigma \left(x_{uk}-x_{uj}\right)\times \left(H_{k\cdot }-H_{j\cdot }\right)-\alpha_{w}W_{u\cdot }, \end{array}$$

(18) $$\begin{array}{c} \nabla H_{i\cdot }=\frac{\partial f\left(\theta \right)}{\partial H_{i\cdot }}=\sigma \left(\frac{x_{ui}-x_{uk}}{1+e^{-m_{uik}}}\right)\times W_{u\cdot }-\alpha_{h}H_{i\cdot }, \end{array}$$

(19) $$\begin{array}{c} \nabla H_{k\cdot }=\frac{\partial f\left(\theta \right)}{\partial H_{k\cdot }}=\sigma \left(\frac{x_{ui}-x_{uk}}{1+e^{-m_{uik}}}\right)\times \left(\frac{1}{1+e^{-m_{uik}}}\right)\times \left(-W_{u\cdot }\right)+\sigma \left(x_{uk}-x_{uj}\right)\times W_{u\cdot }-\alpha_{h}H_{k\cdot }, \end{array}$$

(20) $$\begin{array}{c} \nabla H_{j\cdot }=\frac{\partial f\left(\theta \right)}{\partial H_{j\cdot }}=\sigma \left(x_{uk}-x_{uj}\right)\times \left(-W_{u\cdot }\right)-\alpha_{h}H_{j\cdot }, \end{array}$$

(21) $$\begin{array}{c} \nabla b_{i}=\frac{\partial f\left(\theta \right)}{\partial b_{i}}=\sigma \left(\frac{x_{ui}-x_{uk}}{1+e^{-m_{uik}}}\right)\times \left(\frac{1}{1+e^{-t_{uik}}}\right)-\beta_{h}b_{i}, \end{array}$$

(22) $$\begin{array}{c} \nabla b_{k}=\frac{\partial f\left(\theta \right)}{\partial b_{k}}=\sigma \left(\frac{x_{ui}-x_{uk}}{1+e^{-m_{uik}}}\right)\times \left(\frac{-1}{1+e^{-m_{uik}}}\right)+\sigma \left(x_{uk}-x_{uj}\right)-\beta_{h}b_{k}, \end{array}$$

(23) $$\begin{array}{c} \nabla b_{j}=\frac{\partial f\left(\theta \right)}{\partial b_{j}}=-\frac{1}{1+e^{-m_{uik}}}-\beta_{h}b_{j}, \end{array}$$

where the regularization term is used to void overfitting during model learning and *α*_*w*_, *α*_*h*_, and *β*_*h*_ are hyperparameters.

And thus, we have the updated rules for each variable

(24) $$\begin{array}{c} W_{u\cdot }=W_{u\cdot }-\gamma \nabla W_{u\cdot }, \end{array}$$

(25) $$\begin{array}{c} H_{i\cdot }=H_{i\cdot }-\gamma \nabla H_{i\cdot }, \end{array}$$

(26) $$\begin{array}{c} H_{k\cdot }=H_{k\cdot }-\gamma \nabla H_{k\cdot }, \end{array}$$

(27) $$\begin{array}{c} H_{j\cdot }=H_{j\cdot }-\gamma \nabla H_{j\cdot }, \end{array}$$

(28) $$\begin{array}{c} b_{i}=b_{i}-\gamma \nabla b_{i}, \end{array}$$

(29) $$\begin{array}{c} b_{k}=b_{k}-\gamma \nabla b_{k}, \end{array}$$

(30) $$\begin{array}{c} b_{j}=b_{j}-\gamma \nabla b_{j}, \end{array}$$

where *γ* is the learning rate.

We can find that when the auxiliary feedback of a user has not been observed, the proposed preference assumption in Section 4.1 will be same with the assumption of Bayesian Personalized Ranking (BPR). The algorithm steps of MBPR are depicted in Algorithm 1, where *m* is the number of users and *n* is the number of items.

The pseudocode for model learning is given in Algorithm 1. The user-item observed feedback *S*^Train^ = (*U*, *I*) and auxiliary feedback *AP* are taken as input. First, we split *n* items into three parts. For each iteration, we randomly sample a user *u* (step 1) and then randomly sample items *i*, *j*, and *k* from *P*_*u*_, *AP*_*u*_, and *N*_*u*_ separately (steps 2–4). Specifically, we compute variable gradients according to (17), (18), (19), (20), (21), (22), and (23) (step 5) and then update variables by the gradient descent method (steps 6–12). The auxiliary coefficient *m*_*uik*_ can be computed, respectively, according to the two methods demonstrated in Section 4.3.

The computational time of learning the MBPR model is mainly taken by evaluating the objective function and its gradients against feature vectors (variables). The overall time complexity of MBPR in one iteration is *O*(*d*|*A*| + *d*|*C*|), where *d* is the number of latent factors, *A* is the appointment registration matrix, *C* is the online consultation matrix, and |*A*|, |*C*| refer to the number of observed entries.

## 5. Experiments

In this section, we conduct experiments on the two real-world datasets to evaluate the performance of the proposed method.

### 5.1. Data Sets

We use two real-world datasets in our experimental studies. The Topmd-A dataset is briefly illustrated in Section 3. The website has been combined with high-quality medical resources from 6 hospitals, which are affiliated with Zhengzhou University. By the end of December 2014, it includes 2288 doctors and 38,490 registered users. The main functions provided by the website include Appointment Registration and Online Consultation. Based on the real historical data of the website, we extract data from 20,754 users and 1127 items along with their registration numbers and consultation numbers. The numbers of registration actions and consultation actions are 42,831 and 6735, respectively. Now, the task is interested in a personalized ranked list starting with the doctor who is most likely to be made an appointment with.

In order to demonstrate the generality of the proposed algorithm, experiments are conducted on the datasets from a mobile e-commerce application. The second dataset is coming from Sobazaar mobile shopping app including 17,126 users and 24,785 items. Purchasing data and product-wanted data based on the content interaction are collected. In this situation, “Positive Feedback” represents whether users purchased an item, and the product-wanted data can be considered a variant of “Auxiliary Feedback.” The numbers of purchasing actions and product-wanted actions are accumulated, and the total value is 18,268 and 8916, respectively. Now the task is transformed to predict a personalized ranked list of the items which the user wants to buy next.

The statistics of the two datasets are summarized in Table 2.

### 5.2. Evaluation Metrics

We use the popular ranking-oriented evaluation metrics, Pre*@k* [22, 23], Recall*@k* [14], AUC (area under the curve) [12], MAP (mean average precision) [15], NDCG*@k* [24], and MRR (mean reciprocal rank) [22], to study the recommendation performance of our proposed method in comparison to the baseline works.

#### 5.2.1. *Pre@k*

For each user, the precision of user *u* is defined as Pre_*u*_@*k* = *N*_TP_/(*N*_TP_ + *N*_FP_), where *N*_TP_ is the number of the items which is recommended and user *u* preferred to (true positive, TP), *N*_FP_ is the number of the items which is recommended but user *u* does not prefer to (false positive, FP). And for all users, Pre@*k* is defined as

(31) $$\begin{array}{c} Pre@k=\frac{1}{\left|U^{te}\right|}\sum_{u\in U^{te}}{Pre}_{u}@k. \end{array}$$

#### 5.2.2. *Recall@k*

For each user, Recall@*k* of user *u* is defined as Recall_*u*_@*k* = *N*_TP_/(*N*_TP_ + *N*_FN_), where *N*_FN_ is the number of the items which is not recommended but user *u* preferred to (false negative, FN). And for all users, Recall@*k* is defined as

(32) $$\begin{array}{c} Recall@k=\frac{1}{\left|U^{te}\right|}\sum_{u\in U^{te}}{Recall}_{u}@k. \end{array}$$

#### 5.2.3. AUC

The average AUC statistic is defined as

(33) $$\begin{array}{c} AUC=\frac{1}{\left|U^{te}\right|}\sum_{u\in U^{te}}\frac{1}{\left|E\left(u\right)\right|}\sum_{\left(i,j\right)\in E\left(u\right)}\delta \left({\hat{x}}_{ui}>{\hat{x}}_{uj}\right), \end{array}$$

where *E*(*u*) = {(*i*, *j*) | (*u*, *i*) ∈ *S*^test^∧(*u*, *j*) ∉ (*S*^train^ ∪ *S*^test^)}.

#### 5.2.4. MAP

MAP computes the mean of average precision (AP) over all users in the test set *S*^test^, where AP is the average of precisions computed at all positions with a preferred item

(34) $$\begin{array}{c} {AP}_{u}=\frac{\sum_{i=1}^{Z}pre\left(i\right)\times pref\left(i\right)}{\#\;of\;preferred\;items}, \end{array}$$

where *i* is the position in the rank list, *Z* is the number of retrieved items, and pre(*i*) is the precision of a cutoff rank list from 1 to *i*, pref(*i*) = 1 if the *i*th item is preferred and pref(*i*) = 0 otherwise.

#### 5.2.5. NDCG

The DCG@*k* is defined as

(35) $$\begin{array}{c} DCG@k=\overset{k}{\underset{\ell =1}{\sum }}\frac{2^{\delta \left(d\left(\ell \right)\in I_{u}^{te}\right)}-1}{{\text{log}}_{2}\left(\ell +1\right)}. \end{array}$$

NDCG is the ratio of the DCG value to the ideal DCG value for that user which comes from the best ranking function for the user.

#### 5.2.6. MRR

For each user, the reciprocal rank of user *u* is defined as RR_*u*_ = 1/min_*i*∈*I*_*u*_^*te*^_(*po*_*ui*_), where min_*i*∈*I*_*u*_^*te*^_(*po*_*ui*_) is the position of the first relevant item in the estimated ranking list for user *u*. And then, MRR is defined as

(36) $$\begin{array}{c} MRR=\frac{1}{\left|U^{te}\right|}\sum_{u\in U^{te}}{RR}_{u}. \end{array}$$

### 5.3. Baselines and Parameter Settings

In this paper, the experiments are performed based on *LibRec* (http://www.librec.net/) which is a GPL-licensed Java library for recommender systems, aiming to solve two classic problems: rating prediction and item ranking.

In our experiments, we use 5-fold cross-validation for model learning and testing. Specifically, we randomly split each data set into fivefolds. Fourfolds are used as the training set and the remaining fold as the test set. Five iterations will be conducted to ensure that all folds are tested. And then, the average test performance is reported as the final result.

BPR proposes a pairwise assumption for item ranking and is also a very strong baseline, which is demonstrated to be much better than the well-known pointwise methods (e.g., UGPMF [17], OCCF [15]). Our method is proposed by extending BPR [12] via introducing richer actions, and so, we concentrate our study on comparisons between BPR and our model.

MBPR-1: This method follows the assumption of (6), and the auxiliary coefficient is computed by equation *m*_*uik*_≔*t*_*ui*_ − *t*_*uk*_. The model formulation and learning method are shown in Algorithm 1.

MBPR-2: This method follows the assumption of (6) too, but the auxiliary coefficient *m*_*uik*_ is regarded as one of the model parameters and is iteratively updated using (14) and (15).

For the iteration number *T*, we tried *T* ∈ {30, 100} for all methods. For the number of latent features, we use *d* ∈ {5, 10}. For all experiments, the tradeoff parameters are searched from *α*_*w*_ = *α*_*h*_ = *β*_*h*_ ∈ {0.0001, 0.001, 0.01, 0.1, 1.0}. The NDCG performance on the validation data is used to select the best parameters *α*_*w*_, *α*_*h*_, and *β*_*h*_. And, we can find that the best values of the tradeoff parameters for different methods on different datasets are not the same. The learning rate is used from *γ* ∈ {0.1, 0.01, 0.001}.

### 5.4. Experimental Results and Discussion

**Figure 2:**
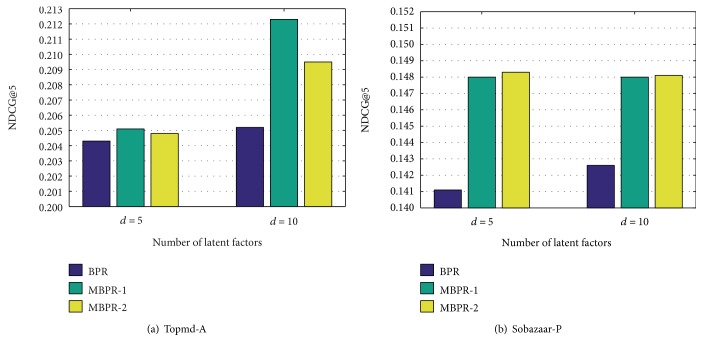
Performance comparison of BPR, MBPR-1, and MBPR-2 on two real-world datasets.

The experimental results of MBPR and other baselines on two real-world datasets are presented in Table 3 and Table 4, and the results of *NDCG* on Topmd-A and Sobazaar-P are shown in Figure 2, from which we can have the following observations:

1. For both datasets, BPR and MBPR are much better than the random algorithm, which shows the effectiveness of pairwise preference assumptions.
2. From the results, it is obvious that our method shows further improvement on all evaluation metrics compared with other algorithms, which demonstrates the effect of injected auxiliary actions. The reason is that BPR model users' preference only based on single kind of positive feedback (e.g., purchasing, viewing, and healthcare reservation), but ignores the fact that auxiliary feedback is very helpful for predicting the users' preference to an item. And so, our method which combines different kinds of pairwise preference over multiple users' actions simultaneously is indeed more effective than the simple pairwise preference assumed in BPR.
3. All models show poor performance on the Sobazaar dataset, the reason we consider is the sparsity of users' positive feedback and auxiliary feedback (which is showed in Table 2). From the percentage of improvements on all the evaluation metrics that MBPR achieves relative to the other models in Tables 3 and 4, it clearly indicates that MBPR shows more significant improvement on Sobazaar-P than Topmd-A. And, this observation demonstrates that our method is specifically helping for the applications in which the data sparseness is more serious.
4. As discussed in Section 4.3, *m*_*uik*_ is computed using two different methods in this paper and a large auxiliary coefficient implies that items have a higher probability of being adopted or preferred by users. We can see that on the two real-world datasets, the performance of MBPR-1 is very close to that of MBPR-2. And one observation from Tables 3 and 4 is that on most evaluation metrics, MBPR-1 performs better than MBPR-2 on Topmd-A, while MBPR-2 performs better than MBPR-1 on Sobazaar-P. Figure 2 clearly shows the same trend in terms of *NDCG*. One possible reason may be that in the context of the Topmd-A dataset for healthcare service, the auxiliary coefficient computed by the first method can indicate the preference distance between the two actions (i.e., appointment registration and online health consultation) more accurately. While in the context of the Sobazaar-P dataset for mobile shopping, the relevance between the users' different actions (i.e., purchasing and product-wanted) is lower. And thus, the two different methods for auxiliary coefficient have little effect on the experimental results in MBPR-1 and MBPR-2.
5. We can find that the two datasets come from different application fields including healthcare service and mobile e-commerce. And thus, the results clearly indicate superior prediction ability of MBPR in various application scenarios.

## 6. Conclusion and Future Work

In this paper, we studied the one-class collaborative filtering problem and designed a novel algorithm called Medical Bayesian Personalized Ranking over multiple users' actions (MBPR). Our novel approach, MBPR, exploits users' different pairwise preference over multiple actions. The two kinds of observed feedback are taken into account simultaneously to improve the predicted performance. Experimental results on two real-world datasets show that MBPR can recommend items more accurately than BPR using various evaluation metrics, and this method is especially suitable for healthcare service recommendation scenarios.

For future work, we are interested in extending MBPR in three aspects: (1) employing an active sampling strategy to select training pairs effectively; (2) studying how to exploit the items' taxonomy information into the MBPR model; (3) exploiting individual healthcare information to model the users' preference order on healthcare services; (4) deploying our model in other real-world healthcare settings to design a more general preference learning solution.

## Figures and Tables

**Algorithm 1:**
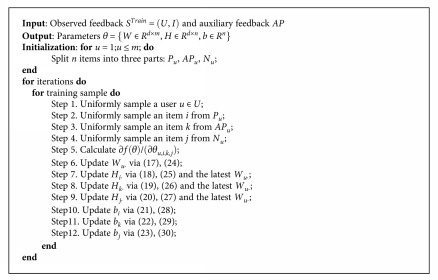
The algorithm of Medical Bayesian Personalized Ranking over multiple users' actions.

**Table 1:** Some notations used in the paper.

Notations	Description
*U* = {*u*}_*u*=1_^*m*^	User set, |*U*| = *m*
*I* = {*i*}_*i*=1_^*n*^	Item set, |*I*| = *n*
*FA*_*u*_ ∈ *I*	Items (preference of observation over user *u*'s one kind action)
*FC*_*u*_ ∈ *I*	Items (preference of observation over user *u*'s auxiliary action)
*F*_*u*_⊆*I*	Items (preference of observation), *F*_*u*_ = *FA*_*u*_ ∪ *FC*_*u*_
${\bar{F}}_{u}\subseteq I$	Items (absence of observation), $F_{u}\cup {\bar{F}}_{u}=I$
*u* ∈ *U*	User index
*i*, *j*, *k*, *j* ∈ *I*	Item index
*P*_*u*_ = {(*u*, *i*)}	Pair set (preference of observation over user *u*'s one kind action), *i* ∈ *FA*_*u*_
*AP*_*u*_ = {(*u*, *k*)}	Pair set (preference of observation over user *u*'s auxiliary action), *k* ∈ *FC*_*u*_
*N*_*u*_ = {(*u*, *j*)}	Pair set (absence of observation), $j\in {\bar{F}}_{u}$
*m*_*uik*_	Auxiliary coefficient, {(*u*, *i*)} ∈ *P*_*u*_, {(*u*, *k*)} ∈ *AP*_*u*_
*t*_*ui*_	The number which user *u* has made to item *i* based on one kind action
*t*_*uk*_	The number which user *u* has made to item *j* based on auxiliary action
*x*_*ui*_	Preference of user *u* on item *i*
*θ*	Model parameters
*W*_*u*·_ ∈ *R*^*d*×*m*^	User *u*'s latent feature vector, *d* is the number of latent factors
*H*_*i*·_ ∈ *R*^*d*×*n*^	User *i*'s latent feature vector, *d* is the number of latent factors
*b*_*i*_ ∈ *R*^*n*^	Item *i*'s bias

**Table 2:** Statistics of the two datasets.

Feature	Topmd-A	Sobazaar-A
Users	20,754	17,126
Items	1127	24,785
Positive feedback	42,831	18,268
Auxiliary feedback	6735	8916

**Table 3:** Recommendation performance of different methods on the dataset from Topmd and row “Improve” shows the percentage of improvements that MBPR achieves relative to the best baseline method.

	Pre@5	Recall@5	AUC	MAP	NDCG	MRR
Random	0.0010	0.0042	0.4989	0.0070	0.1248	0.0076
*d* = 5						
BPR-MF	0.0154	0.0720	0.8384	0.0577	0.2043	0.0602
MBPR-1	0.0154	0.0723	**0.8427**	**0.0584**	**0.2051**	**0.0610**
MBPR-2	**0.0155**	**0.0725**	**0.8427**	0.0580	0.2048	0.0606
Improve	0.64%	0.69%	0.51%	1.21%	0.39%	1.32%
*d* = 10						
BPR-MF	0.0160	0.0749	0.8383	0.0587	0.2052	0.0614
MBPR-1	**0.0172**	**0.0801**	0.8304	**0.0672**	**0.2123**	**0.0707**
MBPR-2	**0.0172**	0.0800	**0.8388**	0.0629	0.2095	0.0661
Improve	7.50%	6.94%	0.05%	14.48%	3.46%	15.14%

**Table 4:** Recommendation performance of different methods on the dataset from Sobazaar.

	Pre@5	Recall@5	AUC	MAP	NDCG	MRR
Random	0.0003	0.0011	0.5035	0.0019	0.1010	0.0023
*d* = 5						
BPR-MF	0.0087	0.0308	0.7203	0.0226	0.1411	0.0292
MBPR-1	**0.0101**	**0.0359**	**0.7464**	0.0263	0.1480	0.0344
MBPR-2	0.0098	0.0351	0.7461	**0.0266**	**0.1483**	**0.0347**
Improve	16.09%	16.56%	3.62%	17.70%	5.10%	18.83%
*d* = 10						
BPR-MF	0.0086	0.0309	0.7290	0.0236	0.1426	0.0308
MBPR-1	0.0098	0.0347	0.7462	**0.0263**	0.1480	0.0344
MBPR-2	**0.0099**	**0.0354**	**0.7464**	**0.0263**	**0.1481**	**0.0345**
Improve	15.11%	14.56%	2.38%	11.44%	3.85%	12.01%
